# Histoplasmosis in Africa: Current perspectives, knowledge gaps, and research priorities

**DOI:** 10.1371/journal.pntd.0010111

**Published:** 2022-02-24

**Authors:** Bright K. Ocansey, Chris Kosmidis, Martin Agyei, Améyo M. Dorkenoo, Olusola O. Ayanlowo, Rita O. Oladele, Tchin Darre, David W. Denning

**Affiliations:** 1 Division of Infection, Immunity and Respiratory Medicine, Faculty of Biology, Medicine and Health, University of Manchester, Manchester Academic Health Science Centre, Manchester, United Kingdom; 2 National Aspergillosis Centre, Wythenshawe Hospital, Manchester University NHS Foundation Trust, Manchester, United Kingdom; 3 Department of Internal Medicine, School of Medicine and Dentistry, Kwame Nkrumah University of Science and Technology, Kumasi, Ghana; 4 Department of Biology and Basic Sciences, Faculty of Health Sciences, University of Lomé, Lomé, Togo; 5 Division of Laboratories, Ministry of Health and Public Hygiene, Lomé, Togo; 6 Department of Medicine, College of Medicine, University of Lagos, Lagos, Nigeria; 7 Department of Medical Microbiology & Parasitology, College of Medicine, University of Lagos, Lagos, Nigeria; 8 Department of Pathology, University Teaching Hospital of Lomé, Lomé, Togo; National Institute for Communicable Diseases, Johannesburg, South Africa, SOUTH AFRICA

## Abstract

**Background:**

Histoplasmosis is a chronic granulomatous disease caused by the thermally dimorphic fungus *Histoplasma capsulatum*. The 2 variants *Histoplasma capsulatum* var. *capsulatum* (Hcc) and *Histoplasma capsulatum* var. *duboisii* (Hcd) causes infection in humans and commonly termed classical or American histoplasmosis and African histoplasmosis, respectively. *Histoplasma capsulatum* var. *farciminosum* (Hcf) affects equines. In recent times, there have been heightened sensitization on fungal infections such as histoplasmosis in Africa, aimed at improving awareness among relevant stakeholders, particularly healthcare workers. This effort is expected to be paralleled with increased detection of both classical and African histoplasmosis, which has remained underdiagnosed over the years. In this narrative review, we describe the current perspectives of histoplasmosis in Africa, identify knowledge gaps, and suggest research priorities.

**Methods:**

A PubMed, Google Scholar, and Africa Journal Online (AJOL) literature search was conducted for studies on histoplasmosis in Africa between 2000 and 2020. Histoplasmosis essays in medical mycology textbooks were also consulted. This narrative review was prepared from the data gathered.

**Findings:**

In the past 2 decades, histoplasmosis in general has seen a relative increase in case detection in some Africa countries, probably attributable to the gradually increasing medical mycology advocacy efforts in Africa. Histoplasmosis cases are dominated by African histoplasmosis mostly in Western and Central Africa, while classical histoplasmosis is more common in Southern and Northern Africa. Although both classical and African histoplasmosis are common in Africa, the latter is more restricted to Africa, and cases outside the continent usually have a travel history to the continent. Despite the clinical and laboratory difference between African histoplasmosis and classical histoplasmosis, it is not straightforward to distinguish them. The typical manifestation of African histoplasmosis is the appearance of lesions affecting the skin, bones, and lymph nodes and unusually linked to human immunodeficiency virus (HIV)/AIDS. By contrast, classical histoplasmosis mostly affects the lungs and is often associated with immunosuppression, mainly HIV/AIDS. The present perspectives of histoplasmosis in Africa highlight unclear details on the true burden, strain diversity, infection route and genetic basis of African histoplasmosis, availability of specie-specific diagnostic tools, and compliance with recommended antifungal therapy. These knowledge gaps represent research questions that require scientific exploration.

**Conclusions:**

Despite a subtle increase in identifying histoplasmosis cases in Africa, it remains underdiagnosed and neglected in some parts of the continent. Increasing awareness and training among healthcare workers, bridging diagnostic and therapeutic gaps, and encouraging more research in Africa are crucial to improve the current perspectives of histoplasmosis in Africa.

## Introduction

Human histoplasmosis is caused by *Histoplasma capsulatum* var. *capsulatum* (Hcc) and *Histoplasma capsulatum* var. *duboisii* (Hcd), which are thermally dimorphic fungi [1,2]. Hcc infection is known as classical or American histoplasmosis due to its established endemism in some parts of North and South America but has a patchy global distribution. On the other hand, its Hcd variant is commonly referred to as African histoplasmosis, owing to its endemism to Africa. It is noted, however, that classical histoplasmosis is also diagnosed on the African continent [3]. Moreover, there have been reported African histoplasmosis cases in Europe, North America, South America, and Asia, but these are usually associated with a travel history to Africa [4–7].

The occurrence of histoplasmosis in Africa is relatively common, although the true disease burden remains unknown, and many more infections are probably missed due to low index of clinical suspicion and unsatisfactory access to diagnostics in endemic areas [2]. African histoplasmosis is generally characterized by lesions affecting the skin, subcutaneous tissues, and lymph nodes. This is in contrast with classical histoplasmosis, which has mainly pulmonary features. Nevertheless, classical histoplasmosis may manifest cutaneous lesions like African histoplasmosis and maybe difficult to distinguish. Unusual cases have been reported to affect internal organs, including the gastrointestinal tract (GIT) [8], the lungs [9], and the brain [10,11], as well as the mouth [12], the eye [13], the penis [14], and vertebrae [15]. Generally, African histoplasmosis has not been associated with any underlying immune deficiencies or genetic defects and is common in immunocompetent patients. However, classical histoplasmosis is frequently associated with advanced human immunodeficiency virus (HIV) disease [15–22]. The cases reported in advanced HIV patients are mostly disseminated, with poor prognosis [22–25].

Clinical, epidemiological, and laboratory characteristics of African histoplasmosis had historically differed from classical histoplasmosis (**Table 1**) but has significant similarities. Over the past few decades, extensive studies have been conducted to improve insights on different aspects of classical histoplasmosis in the western world, providing relevant epidemiological data and simulating further studies to improve diagnosis and treatment [26]. Subsequently, rapid diagnostic methods have been developed, evaluated, and standardized [27–31]. Presently, establishment of surveillance programs and notification of cases in endemic states and territories in the United States [32], addition of *Histoplasma* GM enzyme immunoassay (EIA) to World Health Organization (WHO) essential diagnostics list (EDL) [33], and existence of diagnosis and management guidelines from Infectious Diseases Society of America (IDSA) [31] and Pan American Health Organization (PAHO) [34] are major contributions to the fight against classical histoplasmosis in Europe and the Americas. However, these advancements have not been generally exploited in Africa due to the persistent insufficient awareness and diagnostic and therapeutic challenges [35–38].

**Table 1:** Comparison of epidemiological, clinical, and laboratory characteristics of African and classical histoplasmosis.

	African histoplasmosis	Classical histoplasmosis
**Epidemiology**		
Common areas	Central and Western Africa	Southern and Northern Africa
Transmission	Unknown (inhalation and transcutaneous postulated)	Inhalation
**Clinical**		
Predilection of lesions	Skin, bone, and lymph node	Lung, bone marrow, RET, and adrenal
Auxiliary sites	Rare (GIT and lung)	Common (skin, GIT, and CNS)
Association with HIV	Rare	Common
**Yeast morphology**		
Size of yeast cell (µm)	7 to 15	3 to 5
Cell wall dimension	Thick	Thin
**Management**	Surgical excision, antifungal	Antifungal

CNS, central nervous system; GIT, gastrointestinal tract; HIV, human immunodeficiency virus; RET, reticuloendothelial system.

This narrative review seeks to provide a summary of current perspectives on histoplasmosis in Africa, highlight the knowledge gaps, and identify research priorities needed to improve understanding, increase recognition, and provide better care for patients.

## Methods

PubMed, Google Scholar, and Africa Journal Online (AJOL) were searched for articles on histoplasmosis in Africa from 2000 to 2020 with no language restriction. Published papers were searched using the following keywords and medical subject heading (MeSH) terms: Histoplasmosis, African histoplasmosis, classical/American histoplasmosis, *Histoplasma capsulatum*, *Histoplasma duboisii*, *Histoplasma capsulatum* var. *capsulatum*, and *Histoplasma capsulatum* var. *duboisii* with Africa as well as the 54 sovereign African countries. The Boolean operators “AND” and “OR” were used to combine 2 or 3 keywords and terms. Systematic search for conference abstracts and other “gray literature” was not done mainly because medical mycology meetings have been rarely held in Africa. Additional articles were found through references of initially identified publications (snow balling). All forms of studies including case reports, series, original research, and reviews were considered for inclusion. For case studies, all diagnosed cases demonstrating the presence of Hcc or Hcd in Africans by any laboratory method and accompanying signs and symptoms were included. No specific case definition criteria were employed, largely because the existing expert consensus statements or guidelines are generally not strictly followed or adopted in many African settings and most cases could have been probably excluded. Furthermore, histoplasmosis essays in medical mycology textbooks were also consulted. This narrative review was prepared after going through the data gathered from the above sources.

No ethical approval was required as the study involved analysis of previously published articles.

## Results

Our search found 61 case reports, 9 case series, 5 original articles, and 4 reviews [3,4,7–15,17–26,35,38–90]. These included 20 case reports and 4 case series where diagnosis was made outside Africa and were excluded in discussing the diagnostic and therapeutic capacity of caring for histoplasmosis in Africa.

### Histoplasmosis and *Histoplasma capsulatum* strains in Africa

Histoplasmosis was first reported from Panama by Samuel Darling in 1906; the etiology was Hcc as currently known. Decades later, in 1943, a unique form of histoplasmosis, the Hcd variant, was described by James T. Duncan while investigating an isolate from a case originally from Ghana [2]. In 1952, Hcd was named in honor of Albert Dubois, who provided isolates for further investigations [38]. The initial classification of Hcd as a variant of Hcc was based on phenotypic characteristics, morphology, and geographical distribution [91]. However, recent genetic studies challenge this classification and renders it phylogenetically insignificant. Findings from these studies show that the *Histoplasma* genus contains at least 4 phylogenetic species including an African species that contained *capsulatum*, *duboisii*, and *farciminosum* strains [92–96].

Further phylogenetic analysis of strains within the African clade revealed 3 different groups suggesting the diverse nature of strains on the African continent [95]. None of the identified 3 subgroups that had unique coinciding genotypes were contained in the North America, Latin America, or Eurasia clades. The authors ultimately suggested that the African continent could be harboring more *Histoplasma* species and disease burden than previously reported [95].

The yeast cells of both Hcd and Hcc are uninucleate and share the same mycelial phase, producing macroconidia and microconidia. The latter are the infectious elements reportedly acquired by inhalation. The few studies on the natural reservoir suggest that Hcd and Hcc share a similar ecological niche. In some case studies from Africa, infections were linked to collecting bat guano, living in bat-infested homes, or working with poultry. Recently, Kipyegon and colleagues isolated *H*. *capsulatum* from domestic chicken droppings in Kenya [39].

### Epidemiology

In a review by Oladele and colleagues [3], 470 cases of histoplasmosis were reported in Africa from 1952 to 2017. African histoplasmosis accounted for 61% of cases. A total of 87% of African histoplasmosis cases were from Western and Central Africa. Fewer cases were reported in Eastern and Southern Africa; notably, there were no cases in South Africa despite the significant numbers of classical histoplasmosis cases in the country [3]. A recent review in 2020 estimated that 400 cases of African histoplasmosis alone have been reported from 32 African countries either as case reports or as case series, confirming the predominance of Hcd in Africa [40]. About 29.8% of cases were diagnosed outside Africa, mostly among African indigenes. Two large studies involving single-centered retrospective experiences from the Democratic Republic of Congo and Togo reported 36 (over 6 years) and 17 (over 15 years) cases, respectively [17,18]. *Histoplasma* skin sensitivity surveys continue to be instrumental in defining endemic areas or subclinical histoplasmosis. In Nigeria, a new survey showed *Histoplasma* skin positivity of 4% [41]. However, the endemism of both Hcc and Hcd in Africa and the established cross-reactivity between Hcc and Hcd in histoplasmin skin sensitivity surveys pose a challenge to describing the actual prevalence of the 2 variants.

Different case reviews have reported histoplasmosis to affect more men, with male-to-female ratios averaging 2:1 [17,23,40]. This is also similar for histoplasmin skin surveys [41]. All age groups may be affected, and cases have been reported among those aged between 13 months and 70 years. The reported median ages of case series and reviews range from 21 to 47 years [4,17,40]. Traditionally, histoplasmosis is associated with agricultural workers, carpenters, engagement in outdoor activities, such as chicken runs, collecting bird or bat guano, and living in bat-infested homes [16,17].

Histoplasmosis, especially classical histoplasmosis, commonly overlaps with HIV and tuberculosis (TB) and is a likely to have a significant burden in sub-Saharan Africa. Meanwhile, Africa histoplasmosis seems to have minimal association with HIV, although there are limited data to prove this [15–21] and a few cases have been reported among people with HIV [16,35,36], all of which manifested as disseminated disease. This observation is surprising, considering the strong link between Hcc and HIV infection, especially progressive disseminated histoplasmosis. In uncommon presentations, histoplasmosis has been implicated in immune reconstitution inflammatory syndrome (IRIS) among HIV patients [23,35,87]. Nevertheless, there are cases of disseminated histoplasmosis cases that had occurred in apparently immunocompetent patients [18,20].

Epizootic histoplasmosis may occur among nonhuman primates such as horses, dogs, baboons, and cats. In Africa, high prevalence of equine histoplasmosis is commonly reported in Ethiopia [97]. Although human to animal or animal to animal transmission is not established, a recent case has been attributed to the occurrence of histoplasmosis caused by Hcd in a baboon in America due to the importation of baboons from Senegal [96].

### Infection route

The popular infection route in histoplasmosis is the inhalation of microconidia and subsequent development of pulmonary lesions. Meanwhile, the dominance of cutaneous, subcutaneous, and osseous manifestations of histoplasmosis among Africans highlights some uncertainty. However, it has been widely suggested that Hcc and Hcd share similar infection route with the inhalation phenomenon, but may disseminate to the skin, lymph nodes, or bones by hematogenous or lymphatic spread [8–10]. The transcutaneous route after trauma has also been suggested especially in Hcd and has been implicated in a few case reports on detection of lesion development at sites of acupuncture needle pricks after mud bath [26] and tribal scarification or tattoos [15]. The incubation period of histoplasma yeast is not exactly known, but appearance of lesions has been reported weeks or several years after presumed exposure [46].

Knowledge about the immune response to histoplasmosis, particularly Hcd infection, remains underexploited, and very few studies have been conducted.

### Clinical manifestation

Classical histoplasmosis typically presents as an acute or chronic pulmonary infection, while African histoplasmosis rarely manifests as pulmonary disease but more commonly as an infection of skin, lymph nodes, and bone. However, classical histoplasmosis can also present cutaneous manifestations and may be difficult to distinguish. Disease manifestations vary depending on immune status and the fungal inoculum. In immunocompromised patients, histoplasmosis accounts for significant morbidity and mortality [2]. Pulmonary cases most often mimic TB.

**Fig 1:**
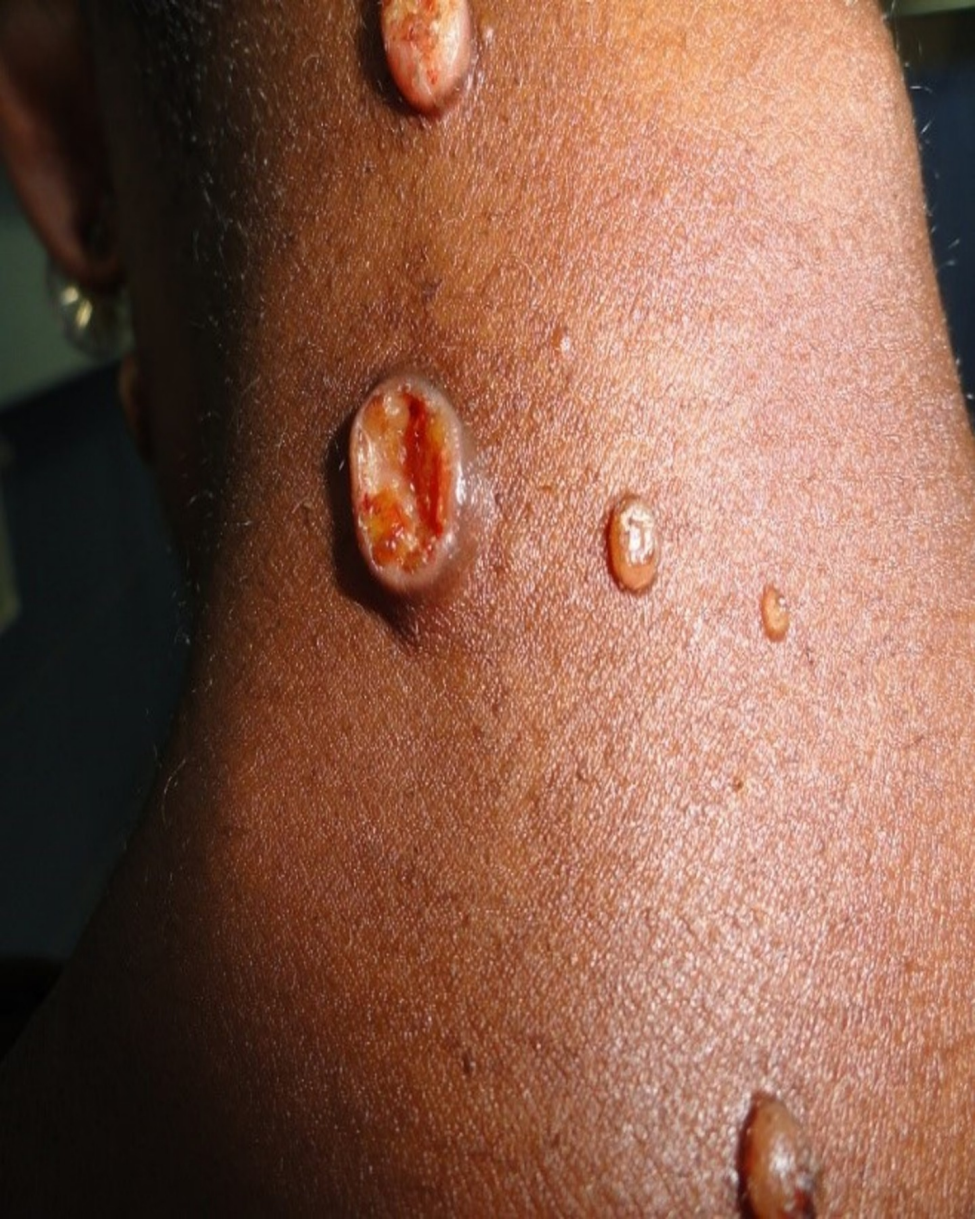
A 36-year-old HIV–negative female with variable sizes of fleshy nodules with umbilication and a well-demarcated scalloping ulcer.

**Fig 2:**
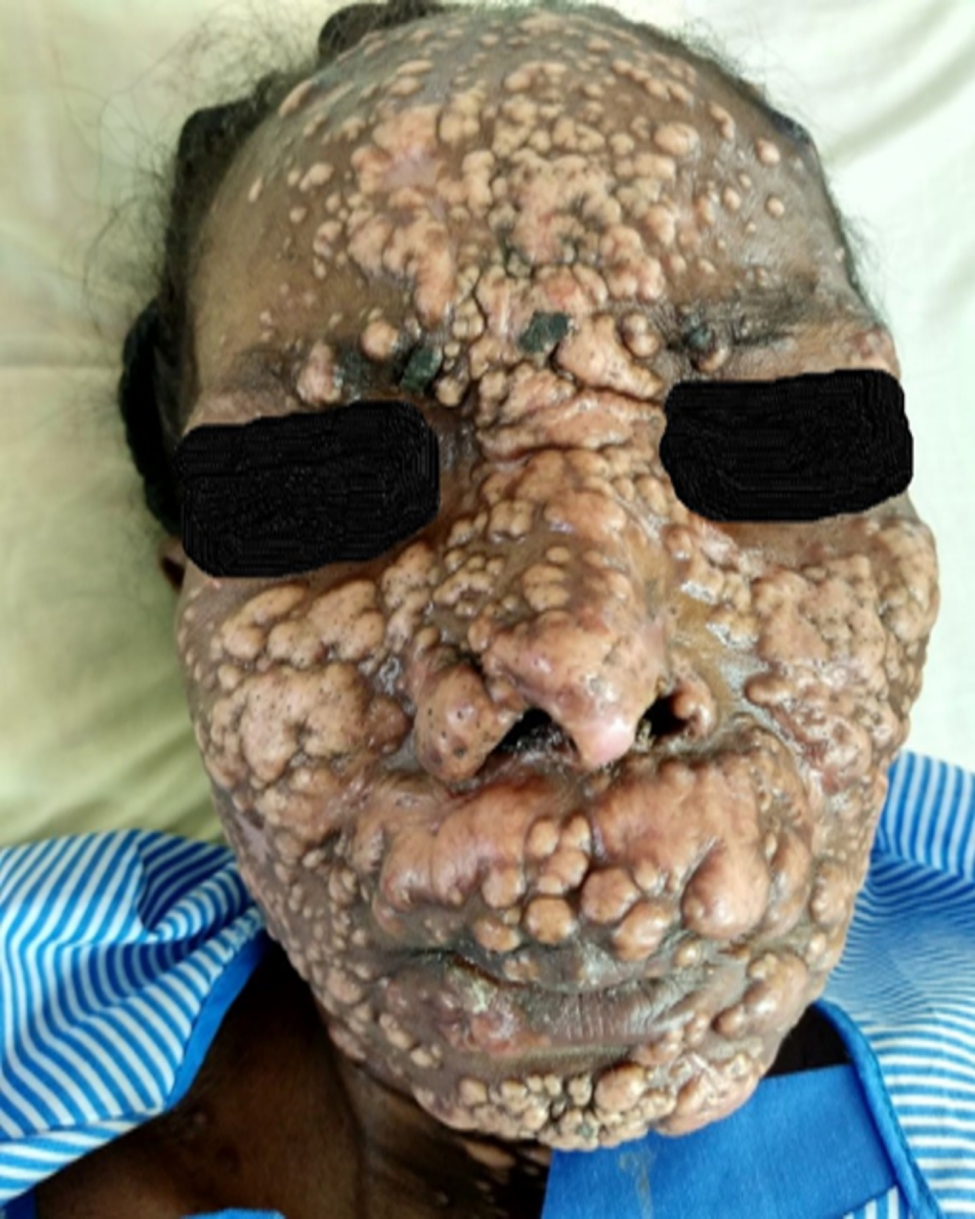
A 43-year-old HIV–positive female with variable sizes of fleshy hypopigmented papules and nodules with coalescing lesions especially on the nose, upper lips, and cheeks with secondary ulcers. *Image courtesy*: *Dr*. *Martin Agyei*.

There are 2 main cutaneous manifestations extensively described in the African continent; localized lesions that may be solitary or confined and scanty with no systemic involvement and disseminated disease presenting as multiple skin lesions in different locations, usually associated with bone, or rarely with deep organ involvement. The diameter of lesions ranges from 0.5 to 8 cm. The cutaneous lesions appearing during histoplasmosis may be papular, nodular, ulcerative, eczematoid, psoriasiform, or circinate lesions (**Figs 1 and 2**) [15]. Lymph node involvement is common, especially around the inguinal, axillary, and cervical regions. The bones are mainly affected in disseminated disease [15]. The skull, ribs, vertebrae, femur, humerus, tibia, and wrist are common targets. Internal body organ lesions are common in disseminated forms of histoplasmosis, but may occur as a solitary lesion [8,9,44,45,49]. Lesions evolve and develop continuously, with new ones appearing as old ones heal, resulting in lesions of different sizes and varied developmental stages [15]. Smaller lesions may resolve without suppuration, while larger lesions develop into a soft cold abscess. Prognosis is good when a solitary lesion is involved [15]. Lymphadenopathy usually accompanies skin lesions; it is mostly superficial, confined to the area of lymph drainage, or generalized in disseminated disease. In very rare cases, internal organs may be affected, mimicking cancers [8–10,45].

The clinical picture of histoplasmosis is not straightforward, and the several forms of cutaneous, subcutaneous, and osseous lesions particularly may look like those of other common skin diseases (**Table 2**). Similarly, infections affecting internal organs may mimic tumor and may require invasive procedures to confirm diagnosis.

**Table 2:** Differential diagnosis of cutaneous, subcutaneous, and osseous manifestations of histoplasmosis.

**Superficial lesion**	
Papules/nodules	Molluscum contagiosum, bromoderma, Kaposi sarcoma, sarcoidosis, common warts, sporotrichosis, leishmaniasis, cryptococcosis, and emergomycosis
Ulcer	Basal cell carcinoma, lupus vulgaris (cutaneous TB), Buruli ulcer, sporotrichosis, leishmaniasis, cryptococcosis, and emergomycosis
Circinate lesions	Tinea corporis and granuloma annulare
Infiltrated plaques	Lichen planus, eczema, psoriasis, and emergomycosis
**Subcutaneous lesions**	
Abscess	Bacterial infection, lipomata, foreign body, guinea-worm abscess, chromoblastomycosis, and cryptococcosis
Multiple granulomas	Mycetoma, blastomycosis, and metastatic malignant neoplasm
**Bone lesions**	
Giant granuloma	Malignant neoplasm, chronic recurrent multifocal osteomyelitis, and blastomycosis
Sinuses	Mycetoma, bacterial osteomyelitis, tuberculous osteomyelitis, actinomycosis, and retained shrapnel

TB, tuberculosis.

### Diagnosis

#### Imaging

Imaging studies are essential when lung or bones are involved and can also be used in monitoring therapy or identifying relapse [26]. X-rays, magnetic resonance imaging (MRI), and computed tomography (CT) have been documented to be useful and employed in Africa [11,14,8,38]. For cases involving the lung, the common radiographic findings are bilateral diffuse opacities, pulmonary infiltrates, nodules, pleural effusion, and nodular cavitation [9,18]. These findings are common in TB, and considering the high TB burden in Africa maybe a recipe for misdiagnosis. CT scan may be necessary to confirm uncertain X-ray findings or demonstrate uncommon features such as disseminated nodules [38].

X-ray findings indicating early bone involvement include reduction in the corticomedullary differentiation of the distal metaphysis, spiculated periosteal reaction, cortical erosion, multiple calcified and noncalcified soft tissue lesions, as well as multiple osteolytic lesions with a sclerotic rim [38]. In subcutaneous lesions, MRI may show lesions that are hyperintense on T2-weighted images and show peripheral rim enhancement. However, bone lesions appear hypointense on T1-weighted images, without rim enhancement after contrast administration.

#### Laboratory diagnosis

Direct microscopy is the simplest, most common, and in majority of the cases, the only available method for the diagnosis of histoplasmosis in several African countries [89]. Smear preparation of exudate or purulent discharge are stained or unstained based on available resources. Used stains include Giemsa, lactophenol cotton blue (LPCB), and Gomori’s methenamine silver (GMS). The use of potassium hydroxide (KOH) also yields good results [24]. Yeast cells of particularly hcd are common in suppurative materials from skin lesions, abscess, discharging sinuses, and bone lesions. Unfortunately, direct microscopy may not confidently detect cases of histoplasmosis as the morphology of *H*. *capsulatum* is similar to other fungi.

The isolation of *H*. *capsulatum* from clinical samples is critical to confirm the diagnosis of histoplasmosis but not generally available or accessible in most laboratories in Africa. Culture is achieved on enriched media such as Sabouraud dextrose agar (SDA) and brain heart infusion (BHI) agar, but its growth is slow, sometimes taking up to 8 weeks. Although it is considered a Class 3 pathogen and requires the use of at least a Class II biosafety cabinet, there is no record of a laboratory-acquired case of histoplasmosis. Macroscopic and microscopic morphology of colonies at room temperature is similar for Hcc and Hcd. Inoculation of primary colonies in appropriate media converts mycelial forms to large yeast forms at 37°C [23]. Suitable media for the conversion include blood or BHI agar supplemented with cysteine or glutamine [23]. *H*. *capsulatum* is usually considered urease negative but may occasionally also be urease positive [7].

The histopathological picture is believed to be identical for classical and African histoplasmosis but may mimic other mycoses blastomycosis, cryptococcosis, and emergomycosis. It is the second common laboratory approach in detecting histoplasmosis in Africa. The histological examination reveals aggregates of multinucleate giant cells containing oval, double-contoured yeast cells. Yeast cells mostly show the “hourglass” or “figure 8” budding, and presence of bud scars is common. The size of the yeast cells is larger, and the walls are thicker in Hcd than in Hcc. The yeast cells may be isolated or sometimes occur in chains of 4 or 5 [35]. Like other fungal infections, staining with only hematoxylin–eosin (HE) may be unsatisfactory, and addition of special stains, periodic acid–Schiff (PAS), and GMS is recommended for better demonstration of yeast cells. Experience is needed to avoid confusing yeast cells of Hcd with other fungi such as capsule-deficient *Cryptococcus* spp. and small variants of *Blastomyces* spp. A positive reaction with mucicarmine is discriminatory for *Cryptococcus* spp.: *Blastomyces* spp. generally has single broad-based buds and is multinucleated on PAS and GMS stains.

The role of immunoassays is currently common in classical histoplasmosis but rarely utilized in Africa. Immunodiffusion and immunofluorescence tests are generally available and occasionally used, but lack consistency, and are often less useful, especially, in immunocompromised patients [42,52]. These assays are not discriminatory between Hcc and Hcd. Galactomannan (GM) and glucan with ß1 to ß4 linkages and ß1 to ß3 linkages are known cell wall and in the mycelial wall constituent of both Hcc and Hcd. The use of GM and beta-D-glucan (BDG) in the diagnosis of African histoplasmosis is unclear, as it has not been extensively used in comparison to classical histoplasmosis. In 2 cases of African histoplasmosis that had *Aspergillus* GM tested, one was positive and the other negative [9,37]. Although it has been suggested that the current commercial *Histoplasma* GM LFA and EIA also detect Hcd, there is currently no evaluation studies to that effect (personal communication).

Molecular identification of Hcc and Hcd are not commonly done in Africa except in few research or tertiary medical settings. Molecular analysis includes panfungal PCR or specific PCR and DNA sequencing of PCR products. These techniques are scarcely available. In a rare clinical experience, Hcd was rapidly detected with PCR and DNA sequencing within 24 hours of admission [36].

Generally, among the 110 cases from both case reports and series of locally diagnosed Hcd and Hcc reviewed, diagnosis was made by histopathology in 96 (87.3%) cases either alone or with direct microscopy, culture, or PCR. Direct microscopy was done in 34 (30.9%) cases and in 20 cases was the only form of diagnosis. Confirmatory diagnosis with culture was done in 22 (20.0%) cases, with positive results in 17, all by phenotypic analysis. Molecular identification was done in only 1 case, on a peripheral blood specimen. Confirmatory of histology diagnosis by PCR and immunohistochemistry was employed in 4 and 12 cases, respectively.

### Management

#### Approaches

Successful management of histoplasmosis relies on appreciating the presence of any underlying condition, affected sites, and the form of disease. Managing an underlying condition is crucial and affects the outcome of targeted treatment [24]. Solitary and scanty superficial lesions may resolve spontaneously or surgical excision, but some may require antifungal therapy [15]. However, for deep lesions and disseminated disease, a combination of surgical removal of lesions and antifungal treatment has proven successful [15,53]. Antifungal therapy is particularly important to reduce the likelihood of relapse and prevent dissemination [23,56].

Presently, treatment data especially for African histoplasmosis consist of individual clinical experiences that have mostly followed the IDSA therapeutic guidelines for histoplasmosis [30] (**Table 3**). The IDSA guidelines recommend either itraconazole or amphotericin B as first-line treatment. The latter preferable in severe and disseminated infection among AIDS patients is mostly not available in several African countries [98]. Although the guideline was originally established for classical histoplasmosis, it has been successfully used in African histoplasmosis cases. However, there are reported clinical experiences with the use of several other antifungal agents in the treatment of histoplasmosis in Africa, mostly itraconazole, fluconazole, and, recently, with posaconazole [37,54]. The regimen varies among clinicians and mostly depends on availability, cost, and, sometimes, ability to manage or monitor tolerance.

**Table 3:** Some recent clinical experience with antifungal therapy in African histoplasmosis.

Country	Form	Regimen	Outcome	Year	Ref
Burkina Faso	D	ITR 800 mg/d	Died	2015	[19]
Chad	D	D-AmB 0.7 mg/kg/d for 15 days then to 1.5 mg/kg/d for 10 months	Cured	2009	[42]
Nigeria	L/thigh	ITR 200 mg/d for 12 months	Cured	2019	[50]
Togo	L/penis	ITR 400 mg/d for 6 months	HCI	2020	[14]
Ghana	D	ITR 200 mg/bd for 47 weeks	CI	2020	[55]
		ITR 100 mg/bd for 2 months and 2 weeks	CI		
		ITR 200 mg/bd for 2 months	Cured		
Togo	D	ITR 400 mg/d for 4 months		2018	[56]
		FLC 450 mg/d for 4 months	CI		

D, disseminated; D-AmB, deoxycholate amphotericin B; FLC, fluconazole; HCI, high clinical improvement; ITR, itraconazole; L, localized.

#### Outcomes

Most cases of acute histoplasmosis in immunocompetent individuals tend to resolve spontaneously [2]. Similarly, most patients with histoplasmosis, particularly those with a single or few cutaneous lesions, recover completely. Several successful treatment regimens have been reported for different antifungal agents in Africa. The dosage and duration vary, and outcomes are inconsistent (**Table 3**). Cutaneous lesions may evolve and develop continuously, with the emergence of breakthrough lesions where new ones appear as old ones heal during antifungal therapy [15]. This may not require therapeutic adjustment because new lesions subsequently yield to ongoing treatment. Relapse is common when antifungal therapy is halted especially in disseminated cases. Disseminated histoplasmosis, especially in HIV patients, has a terrible outcome in Africa. The case fatality rate is estimated to be around 23% for Hcd and about 50% for Hcc [52,98].

### Knowledge gaps and research priorities

Histoplasmosis is gradually being appreciated in the African continent, although awareness can be further improved. This summary sought to highlight the perspectives of histoplasmosis in Africa and identifies multiple avenues for scientific exploration, particularly by African researchers:

- The number of species contained in the genus *Histoplasma* remains uncertain, probably with several cryptic species. This requires further research to establish postulates suggesting Hcd as phylogenetic species and not a variant. Unraveling the species confusion within the *Histoplasma* genus may contribute to the development of species-specific diagnostics as both Hcd and Hcc are common in Africa. Additionally, pursuing further studies to understand the phylogenetic structure of the 3 subgroups of the African clade in establishing the genetic diversity of African strains of both Hcc and Hcd.
- The true burden of histoplasmosis is currently unknown, and adequate mapping is lacking. There is a need to probably institute active localized surveillance programs in endemic areas, particularly in previously suggested epicenters in Transvaal and Cape Province, South Africa [35], Kimpese, Democratic Republic of the Congo [18], Ogbunike, Nigeria [35], and Brazzaville, Republic of Congo [99], and rural areas with potentially high risks. Nearly all cases of histoplasmosis reported in the literature are diagnosed in tertiary medical centers in urban cities, and patients sometimes need to travel long distances. Organizing awareness programs and diagnostic workup network is important to increase clinical suspicion among healthcare workers and encourage community inhabitants to engage with healthcare facilities.
- The role of cell-mediated immune defects in worsening the outcome of persons with HIV or with TB coinfection is not clear. Considering the high burden of HIV/AIDS and TB in most African countries, it is important to investigate the exact relationship between histoplasmosis, HIV, and TB.
- The genetic basis of African histoplasmosis is undetermined, and the reason why it is common in Africans remains unanswered. There is a need to broaden the understanding of genetic susceptibility factors associated with African histoplasmosis. Conducting gene association mapping studies maybe important to identify susceptible loci in African histoplasmosis patients.
- The infection route of African histoplasmosis is another rarely exploited aspect in the efforts to understand the disease. It is still unclear how people become infected, and there are several challenging questions about the various stages leading to the development of extensive cutaneous manifestation dominant among Africans. Screening and culturing of fungi from habitats are needed in supposedly endemic areas to properly define the environmental niche of the fungi. There is scarce evidence to support inhalation and inoculation via transcutaneous routes as the main modes of transmission. The role of animal reservoirs as intermediate hosts should be investigated in cattle, chicken, sheep, goats, and dogs in endemic areas where people live in proximity with these animals. All these are important in developing control measures.
- Confidently discriminating Hcd from Hcc is a challenge in areas where both are significantly reported particularly in Africa. Moreover, recent studies have questioned the reliability of the classical differentiation approach based on cell size in tissue (histopathology) or in vitro (culture). It is important to develop and increase access to accurate diagnostic tool that can rapidly and reliably detect Hcc and Hcd especially in endemic areas where access to histopathological and advanced mycology facilities is inadequate [100]. The subtle antigen variation in Hcd and Hcc must be exploited to develop species-specific, fast, and reliable tests. Also, considering the relevance of culture in obtaining isolates for epidemiological and antifungal susceptibility analysis, there is a need to also develop biochemical methods to distinguish Hcd from Hcc isolates.

The current therapeutic guidelines or recommendations renders management of histoplasmosis especially in severe, disseminated cases and in children a dilemma to clinicians as recommended antifungal drugs is not commonly available. Therefore, there is a need to evaluate other common antifungal drugs as alternative treatment options.

## Conclusions

Histoplasmosis is an underdiagnosed infection that affects both immunocompromised and apparently healthy individuals and is gradually gaining recognition on the African continent. To better understand and improve diagnosis and management, especially in endemic areas, several principal questions must be answered by undertaking strategic advocacy and research efforts. This narrative of the current perspectives of histoplasmosis reveals mainly diagnostic and therapeutic deficiencies. The present knowledge gaps call for critical research. More importantly, increasing awareness, particularly, among healthcare workers and researchers in Africa is paramount. This requires continuously improving recognition in the African community. Histoplasmosis must also be considered for inclusion in WHO neglected tropical disease (NTD) category of “other fungal diseases” alongside mycetoma, chromoblastomycosis, and sporotrichosis.

**Key learning points**

- There is an improved awareness and case detection of histoplasmosis in some parts of Africa, but it remains relatively underdiagnosed.
- Current data on histoplasmosis in Africa are dominated by case reports and reviews.
- Data on true burden, genetic diversity of African strains, infection route and genetic susceptibility factors for African histoplasmosis, and development and evaluation of specie-specific diagnostic tools are limited.
- Diagnostic and therapeutic advances seen in the western world is insufficiently available in Africa.
- Advocacy and training of healthcare workers must be broadened on the continent.

**Top five papers**

1. Oladele RO, Ayanlowo OO, Richardson MD, Denning DW. Histoplasmosis in Africa: An emerging or a neglected disease? PLoS Negl Trop Dis. 2018 Jan 18;12(1):e0006046.
2. Develoux M, Amona FM, Hennequin C. Histoplasmosis caused by Histoplasma capsulatum var. duboisii: a comprehensive review of cases from 1993 to 2019. Clin Infect Dis. 2020 Sep 4.
3. Gugnani HC. Histoplasmosis in Africa: a review. Indian J Chest Dis Allied Sci. 2000 Oct 1;42(4):271–8.
4. Updates in the Language of Histoplasma Biodiversity. mBio. 2018 Jul 5;9(3):e00181–18.
5. Mandengue CE, Ngandjio A, Atangana PJ. Histoplasmosis in HIV-infected persons, Yaounde, Cameroon. Emerging infectious diseases. 2015 Nov;21(11):2094.
